# Effective Project Management of a Pan-African Cancer Research Network: Men of African Descent and Carcinoma of the Prostate (MADCaP)

**DOI:** 10.1200/JGO.18.00062

**Published:** 2018-09-27

**Authors:** Emeka Odiaka, David W. Lounsbury, Mohamed Jalloh, Ben Adusei, Thierno Amadou Diallo, Papa Moussa Sene Kane, Isabella Rockson, Vicky Okyne, Hayley Irusen, Audrey Pentz, Ifeoluwa Makinde, Olalekan Hafees Ajibola, Lindsay Petersen, Jo McBride, Desiree C. Petersen, Sunny Mante, Ilir Agalliu, Akindele Olupelumi Adebiyi, Olufemi Popoola, Edward Yeboah, James E. Mensah, Ann Hsing, Pedro Fernandez, Oseremen Aisuodionoe-Shadrach, Maureen Joffe, Elvira Singh, Serigne Magueye Gueye, Yuri Quintana, Brian Fortier, Timothy R. Rebbeck, Caroline Andrews

**Affiliations:** **Emeka Odiaka, Ifeoluwa Makinde, Akindele Olupelumi Adebiyi, and Olufemi Popoola,** University College Hospital, Ibadan; **Olalekan Hafees Ajibola and Oseremen Aisuodionoe-Shadrach,** University of Abuja; **Oseremen Aisuodionoe-Shadrach,** University of Abuja Teaching Hospital, Abuja, Nigeria; **David W. Lounsbury and Ilir Agalliu,** Albert Einstein College of Medicine, Bronx, NY; **Mohamed Jalloh, Thierno Amadou Diallo, Papa Moussa Sene Kane, and Serigne Magueye Gueye, Hôpital Général de Grand Yoff,** Institut de Formation et de la Recherche en Urologie et de la Santé de la Familliale, Dakar, Senegal**; Ben Adusei and Sunny Mante,** 37 Military Hospital, **Ghana; Isabella Rockson, Vicky Okyne, Edward Yeboah, and James E. Mensah,** Korle-Bu Teaching Hospital, and University of Ghana, Accra, Ghana; **Hayley Irusen and Pedro Fernandez,** Stellenbosch University and Tygerberg Hospital; **Lindsay Petersen, Jo McBride, and Desiree C. Petersen,** Centre for Proteomic and Genomic Research, Cape Town; **Audrey Pentz, Elvira Singh, and Maureen Joffe,** University of the Witwatersrand, Johannesburg, South Africa; **Ann Hsing,** Stanford University, Stanford, CA; **Yuri Quintana,** Beth Israel Deaconess Medical Center; **Brian Fortier, Timothy R. Rebbeck, and Caroline Andrews,** Dana-Farber Cancer Institute; **and Timothy R. Rebbeck,** Harvard TH Chan School of Public Health, Boston, MA.

## Abstract

**Purpose:**

Health research in low- and middle-income countries can generate novel scientific knowledge and improve clinical care, fostering population health improvements to prevent premature death. Project management is a critical part of the success of this research, applying knowledge, skills, tools, and techniques to accomplish required goals. Here, we describe the development and implementation of tools to support a multifaceted study of prostate cancer in Africa, focusing on building strategic and operational capacity.

**Methods:**

Applying a learning organizational framework, we developed and implemented a project management toolkit (PMT) that includes a management process flowchart, a cyclical center-specific schedule of activities, periodic reporting and communication, and center-specific monitoring and evaluation metrics.

**Results:**

The PMT was successfully deployed during year one of the project with effective component implementation occurring through periodic cycles of dissemination and feedback to local center project managers. A specific evaluation was conducted 1 year after study initiation to obtain enrollment data, evaluate individual quality control management plans, and undertake risk log assessments and follow-up. Pilot data obtained identified areas in which centers required mentoring, strengthening, and capacity development. Strategies were implemented to improve project goals and operational capacity through local problem solving, conducting quality control checks and following compliancy with study aims. Moving forward, centers will perform quarterly evaluations and initiate strengthening measures as required.

**Conclusion:**

The PMT has fostered the development of both strategic and operational capacity across project centers. Investment in project management resources is essential to ensuring high-quality, impactful health research in low- and middle-income countries.

## INTRODUCTION

Effective health-related research relies on effective project management to ensure that the required tools, techniques, strategies, and systematic processes have been implemented and adhered to to enable the production of quality data that can affect disease prevention and control, as well as policy needs, considering the local and national political and social environment.^1^ Integrating project management capacity into health system–strengthening activities for low- and middle-income countries (LMICs) has the potential to improve the health of the population served^2^; however, the development and evaluation of the tools and skills for effective project management are rarely available in the research undertaken in an African setting.^3^ To achieve impactful research in LMICs, there is a need for training and education for individuals working at all levels, including project managers (PMs), not just among high-level research leadership.^4^

Health research initiatives in LMICs can help foster capacity building at the local level; however, such efforts are often limited to specific, limited aims that restrict opportunities to build sustainable partnerships and relationships among LMIC partners to positively affect health outcomes.^3^ Developing project management capacity into activities that can broadly strengthen health systems in LMICs has the potential to improve target organizations’ performance and the health of the population served.^2^ Equally important is moving beyond short-term, project-specific objectives focused on a particular disease or population to envision ways to leverage project resources to strengthen the entire health system.^5,6^

Health research capacity consists of two basic components, strategic capacity and operational capacity.^7^ Strategic capacity in research is defined by each project’s specific aims, objectives, research questions, and research design. As research projects are implemented, it is reflected in the ability to problem solve and retain fidelity to the stated aims. Operational capacity comes from supporting project-specific policies and procedures, which often include pilot studies and assorted quality control checks and may use electronic management systems to help track project progress.

Building health research capacity can be achieved over time when there is sufficient investment in and a commitment to improving practices^7^; however, the demands on any contemporary health system, whether situated in a high-income country or an LMIC, often include human and financial resource constraints, aging or obsolete infrastructure, and ever-changing policies and regulations striving to accommodate the latest clinical practice guidelines. To achieve successful research output, complex organizations, such as hospitals and clinics, must continuously transform and adapt to new circumstances. Organizations that have this capacity have been dubbed learning organizations^8^ and have comparatively greater success in improving their effectiveness and efficiency.^9-11^ Whereas the concept of cultivating a learning organization has been applied in many different types of organizations, it is a relatively new idea in health systems, particularly in health systems that serve LMICs.^12^

A learning organizational framework lends itself to developing management practices that foster effectiveness at all levels, appreciating the local as well as the global sociocultural context in which a given project operates.^13,14^

Prostate cancer is the leading cancer in Sub-Saharan African men, and men of African descent have higher prostate cancer frequency, severity, and death than men of other races, yet little is known of its etiology.^15^ Therefore, dedicated resources are needed to try to elucidate the causes. Here, we describe a multicenter, international, hospital-based, case-control study of genetic epidemiology of prostate cancer in men of African descent and the creation of a project manager toolkit (PMT; Table 1).^16^ This study aimed at facilitating the standardization of protocols and project management for the study, with ongoing monitoring and evaluation using guidelines recommended by NIH,^17,18^ as well as developing strategic and operational capacity for cancer research for each participating center. The study, to be conducted over a 5-year period, was awarded to the Dana-Farber Cancer Institute (T.R., principal investigator [PI]) and the Men of African Descent and Carcinoma of the Prostate (MADCaP) network^19^ by the National Cancer Institute.

**Table 1:**
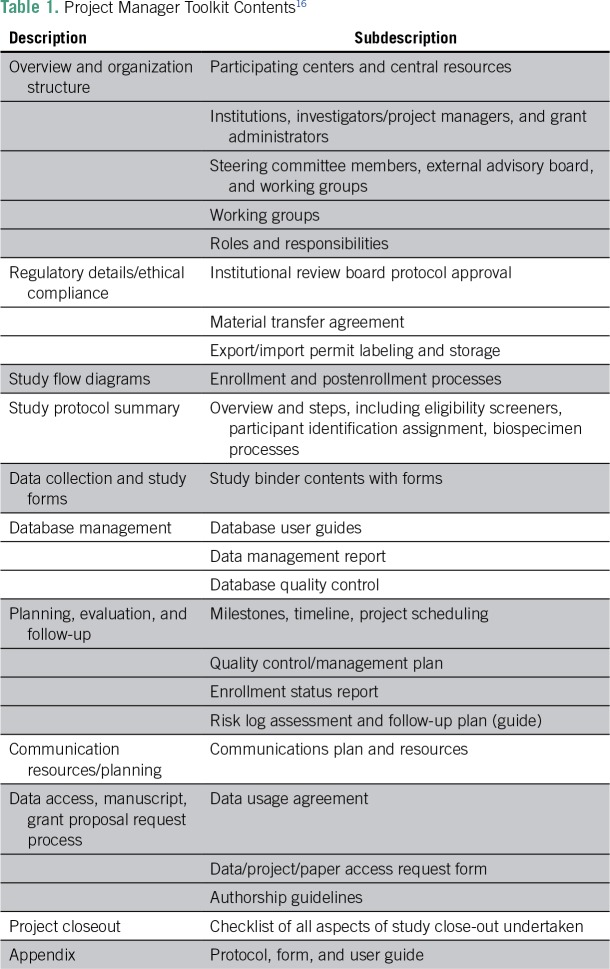
Project Manager Toolkit Contents^16^

## METHODS

### Multicenter Collaborative Structure

**Fig 1:**
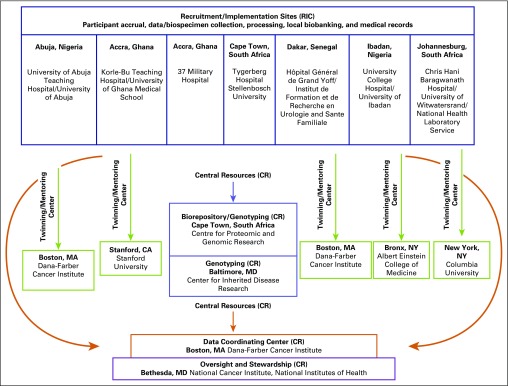
Organizational Chart. Central resources (CRs): Center for Proteomics and Genomics Research, Cape Town, South Africa; Dana-Farber Cancer Institute, Boston, MA; Center for Inherited Disease Research and the Intramural Program of the National Cancer Institute, Bethesda, MD. Twinning/mentoring centers: Dana-Farber Cancer Institute, Boston, MA; Stanford University, Stanford, CA; Albert Einstein College of Medicine, Bronx, NY; Columbia University, New York, NY.

The MADCaP research study includes seven participating recruitment and implementation centers (RICs) from four African countries—Nigeria, Senegal, Ghana, and South Africa—linked to four twinned/mentoring centers (TMCs) located in the United States and four central resources (CRs) participating as an international team. A steering committee and external advisory board provided overall oversight and stewardship of the MADCaP study (Fig 1). The TMC partnership serves to advise, support, and promote the research being conducted at the twinned LMIC center, transferring expertise, skills, and knowledge with the goal of establishing a long-term collaboration.^20^

**Fig 2:**
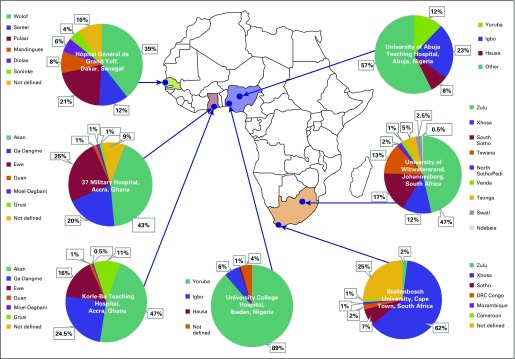
Population breakdown of the black African cohort enrolled at the African recruitment and implementation centers. Breakdown of ethnicity from patients and controls enrolled by the seven recruitment and implementation centers. University of Abuja Teaching Hospital Other category includes Afo, Baju, Bini, Busi, Ebira, Ebu, Edo, Ego, Esan, Ewe, Fulani, Gbagyi, Gwandara, Gwari, Idoma, Igala, Ijaw, Ikuku, Ishan, Isoko, Kahoma, Koro, Mada, Owhan, Tarok, Tiv, and Ukpela.

Each RIC identifies and recruits participants with prostate cancer and age-matched controls—5-year age groups—from local hospitals/clinics, collects epidemiologic questionnaires and biospecimens (blood or saliva), and performs DNA extraction and storage. Eligible participants and controls provide data on demographics (Fig 2), anthropometrics, physical activity and lifestyle, medical history, and family history. Survey data are entered with medical record abstracted data into a secure database (https://www.datstat.com) created for the study. The PMT was primarily designed to support these study operations.

The Project Manager Working Group (PMWG) was formed at the start of the study to support the design and implementation of the PMT. The PMWG included representation from each RIC, TMC, and CR participating as an international team to develop, implement, and evaluate the toolkit.

Each RIC was composed of a team that included physicians, project managers, research coordinators, phlebotomists, laboratory specialists, grant managers, institutional review board managers, database managers, and other study staff assembled by the local PI. An electronic survey^21^ was conducted to further understand emergent patterns of roles and responsibilities undertaken by RIC staff. A data coordinating center was established at the Dana-Farber Cancer Institute as a central hub for all data collection, processing, quality control, and storage for common study data. The Centre for Proteomic and Genome Research served as a resource for facilitating RIC infrastructure to collect biospecimens, perform DNA extractions, conduct quantitative and qualitative analyses, and facilitate the initial storage of extracted DNA and final shipment to genotyping centers. Using a common set of protocols developed for the network, the PM at each center was responsible for managing the study under guidance and direction of the PI to ensure that study objectives, milestones, and deliverables were reached.

### PMT Development and Implementation Processes

**Fig 3:**
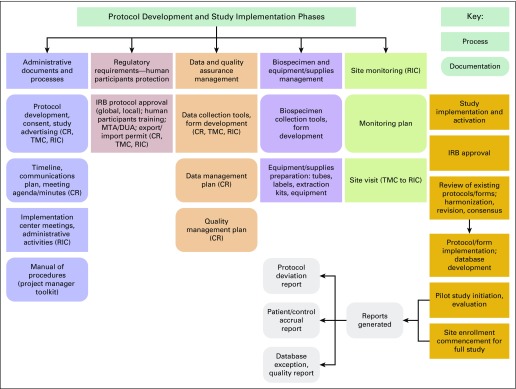
Overview of study protocol development and implementation phases. CR, central resource; DUA, data use agreement; IRB, institutional review board; MTA, mutual transfer agreement; RIC, recruitment and implementation center; TMC, twinning/mentoring center.

Through an iterative collaborative development initiative, standardized protocols, data instruments, and recommended practices were drafted and shared across participating centers. PMT contents were systematically developed over the study’s start-up period, designed to accommodate the organizational structure at African Sub-Saharan participating study centers (Fig 3), and organized to support the key elements of study management (Table 1).

The following processes were used to develop and implement the PMT:

Timeline charting: A Gantt chart categorizing important tasks by anticipated start and completion times was shared with PMs. Adherence to completion times was monitored closely during the study’s start-up phase, understanding that challenges to keeping to the timeline would vary across centers (Data Supplement).
Training: Videos, training manuals, individual Web meetings, and in-person group training were developed by CR teams.^22^
Data processing support: To facilitate best practices for data entry, management, and quality control, a mechanism for electronic communication was incorporated into the PMT. Guidelines designating responsibility for the preparation and sharing of data, processing information, frequency of data distribution, and data dissemination were drafted and disseminated to PMs and PIs.
Promoting online communication: A Web-based communications platform,^19^ built on the Alicanto software (http://www.alicantocloud.com), was created for MADCaP network members to help build relationships and facilitate timely communication to support the development of community of practice where participants learn from each other and there is a more equal relationship between experts and learners, as opposed to the traditional model of teachers and students.^23^ It serves as a centralized location for all study information, including research protocols, instruments, progress reports, and research team member profiles, to enhance communication strategies. Information exchanges took place in person and via frequent, coordinated individual center and consortia Web conferencing using the communications tool, Zoom (https://zoom.us). This software works well in low-bandwidth conditions and was integrated into the platform’s architecture along with calendaring capabilities for ease of user project activities.
Dissemination and archiving of project management information: Timely distribution of meeting agendas and minutes highlighting progress and action items served to document study progress. Over time, these materials formed an archive that is centrally accessible through the MADCaP communication platform.
Site visits: Representatives from TMCs and CRs conducted site visits to each RIC to evaluate the local context and compliance to standardized study protocols and to provide guidance and recommendations.
Formation of additional working groups: As the study progressed, it became clear that additional working groups were needed to develop and implement specific study activities. Protocol and data elements and biospecimen and biobanking working groups were established at study initiation to develop, standardize, and finalize the protocols and data instruments for use in the multicenter study. Each RIC undertook a pilot study over a defined period to assess the protocols, survey delivery, and documenting challenges/successes encountered. Conducting frequent conference calls among RICs provided an opportunity to examine and report project flow weaknesses/strengths, recording local modifications needed for successful study implementation. These were shared back to the larger group for additional discussion, which led to document revisions for use in the main study, a core component of the PMT, and to ensure comprehensive study compliance. Additional working groups were established and will continue to form as the life cycle of the study moves through different phases and additional projects branch off.

## RESULTS

### Project Management Roles and Responsibilities

Data obtained from conducting the survey of project management roles and responsibilities helped to further understand emergent patterns of roles and responsibilities undertaken by RIC staff, yielding 51 individual respondents from seven centers with varying professional and technical backgrounds, which clustered into five major categories as follows:

Project management: meeting organization/calendaring, overall study facilitation, and data entry into databases.
Finance and contracts: invoicing and reconciliation, financial management, grant administration, and budget development.
Participant recruitment and interviewing: subject consent and interview, patient/control eligibility screening, and form labeling/storage of participant folders.
Medical record review and abstraction: retrieval of paper or electronic patient information.
Biotechnology: phlebotomy and sample labeling, biospecimen delivery to the laboratory for DNA extraction, quality control measures, and storage and shipment of DNA.

### Monitoring, Formative Evaluation, and Pilot Testing of the PMT

PMs from RICs were asked to share their experiences during the pilot phase of the study, describing eligible participants, percent declining enrollment, number of visits required to complete enrollment, challenges encountered, and experiences gained (Table 2). Feedback from this initial presentation revealed the following three focus areas that PMs used to monitor progress throughout the research study.

**Table 2:**
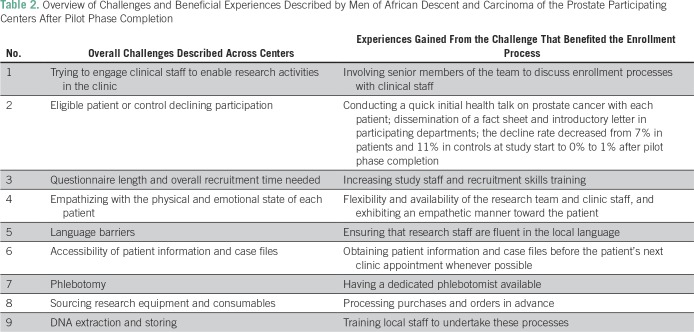
Overview of Challenges and Beneficial Experiences Described by Men of African Descent and Carcinoma of the Prostate Participating Centers After Pilot Phase Completion

#### Data quality.

Measures were implemented to monitor quality, reliability, accuracy, and completeness of the data being collected and entered into the study database to provide a mechanism for feedback to database administrators. In addition, a comparison between original data entry at each RIC and duplicate data entry was performed on a subset—10% of random samples—of enrolled participants. Second data entry was performed by RIC data entry staff and results were circulated to CR centers. The PM was responsible for receiving discrepancy reports and ensuring that differences were resolved.

#### Quality control and management.

A quality management resource plan was established to obtain an ongoing assessment of quality indicators.^24^ The RIC PM completed sections of this plan related to the consent process and signature; protocol compliance, form completion, labeling, and appropriate storage; biospecimen handling and compliance with laboratory protocols; database entry; and institution-specific training and training logs.

#### Project risk assessment and follow-up plan.

To understand, maintain, and improve performance throughout the study lifecycle, this plan^25^ was established as a stepwise process in which risk assessment was recorded on a numeric scale from 1 to 5. The RIC PM performed an initial risk assessment, following standardized guidelines, considering the risk impact that each main study category might have on the study process success action and outcome. Assessment of the impact on project risk was scaled from 1 (minimal) to 5 (very severe), initiating action/mitigation as needed and designating an individual to undertake the required task. On subsequent assessments, a status category would be completed that defined whether the effect had been averted, was reducing, or was on the rise (Table 3).

**Table 3:**
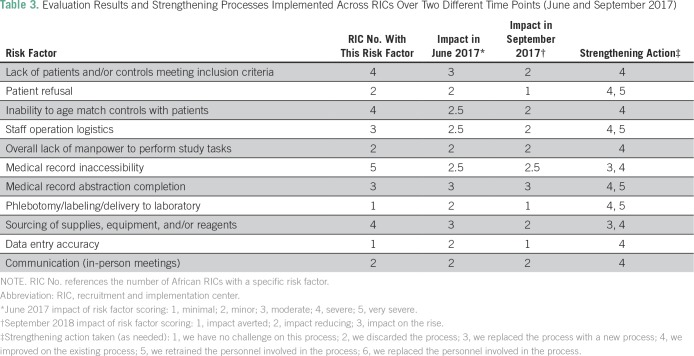
Evaluation Results and Strengthening Processes Implemented Across RICs Over Two Different Time Points (June and September 2017)

Results of the first evaluation conducted by RIC PMs using the PMT monitoring and evaluation tools during the main study period revealed several areas in which challenges existed with varying impact factors. The PM tasked a study team member to initiate action to achieve an improvement plan and solution. These included:

Recruitment/accrual targets: Several centers initially fell below their accrual goal. Accrual barriers included insufficient eligible participants, language barriers, country-wide hospital strikes shutting down operations, patient costs for obtaining biopsies required for eligibility inclusion, delays in sourcing necessary diagnostic supplies, and other staff/institutional logistics. Solutions included extending recruitment to additional hospitals and clinics within the catchment area, recruiting interpreters, and increasing communication channels and relationships between hospital and research staff.
Protocol noncompliance: One center reported protocol completion noncompliance. One participant failed to complete the consent process because of time constraints and three others declined consent. This center found that offering a small token of appreciation of travel vouchers and/or snacks and having the caring physician initially introduce the study to his patient enhanced protocol compliance. One center required multiple participant visits to complete enrollment, thus maintaining a constant line of communication with the research participant and reminding him of upcoming hospital visits via text message enhanced his research study participation.
Data management: Two centers experienced difficulties initially accessing and navigating the online research database, administered by DatStat (www.datstat.com). This was overcome via focused one-on-one training initiatives with the data coordinating center and the installation of a remote data collection module that permitted offline data entry. This allowed the center to send or sync collected data to the central online database when a stable Internet connection existed.

Metrics were subsequently devised and incorporated as reportable factors for the quarterly assessment/analytical report of the PMT.^26^ Main study evaluation was subsequently undertaken on a quarterly basis with strengthening processes initiated for the last 6 months described in Table 3.

## DISCUSSION

We have described a PMT created expressly to support front-line MADCaP local project staff in establishing and conducting a multicenter study of the genetic epidemiology of prostate cancer in African men. PMs need training for the specific research aims, but to work optimally they also need skills and experience in risk analysis, setting priorities, planning, budgeting, human relations, team building, and incentivizing performance.^7^ The PMT steps away from a top-down approach in which donors and external experts exclusively inform the study protocol with limited input from local stakeholders and seeks to engage and empower local stakeholders through bottom-up strategies that more often help create and sustain local organizational capacity.^27^ To be effective, the toolkit guides each organization to effectively participate in the study while continually learning about and adapting to their local realities. As a continuous active learning model, this PMT guides evaluation phases that will be developed, revised, strengthened, and extended throughout the project lifecycle. The PMT also helps to achieve our aim of enhancing the capacity of MADCaP’s African organizations by emphasizing local capacity building from the ground up and strengthening center interactions.

Successful research outcomes are dependent on the environment and organizational structure in which the research study operates. Lack of planning, poor quality control, inadequate risk management, inefficient organizational structure, and breakdowns in communication have been associated with long-term project failure.^28^ Key challenges in the project management of multi-institutional, multicenter, clinical research studies include project team communication, staff training, efficient and accurate record keeping and data integrity, research staff access to clinical areas, staff turnover, participant recruitment and retention, and navigation of institutional review boards and other regulatory bodies.^29^ Site-specific adaptations are also key to a successful outcome, particularly when studies are conducted in multiple countries. In the current study, one center created a cross-center intervention committee to review and approve initial protocols, proposing adaptations and the implementation of intervention components throughout the study lifecycle. All stages of the development of the PMT were the result of intensive interaction with the center. This interaction ensured the appropriateness and sustainability of its contents to these resource-limited settings. Intervention adaptation was essential for scenarios that were unforeseen during the design and study initiation phases. Such enhancements increased the value of the study both locally and globally.

Formative evaluation of the PMT indicates that it has fostered the development of both strategic and operational capacity across participating study centers. Implementation and preliminary use of the PMT monitoring and evaluation module permitted the measurement of improvement for defined processes that will continue to develop and be adapted throughout the project lifecycle. This remains essential for all aspects of the study, especially in developing confidence and rigor toward the different tasks and capacity building. Within and across centers, the PMT promoted adaptive change and accountability, which facilitates project success. Through use of the toolkit, common expectations and shared understanding about goals and objectives led to the dissemination of innovative ideas and processes as well as to scaling up of effective interventions, which can bring additional resources for health systems development. Where local research capacities are developed, improved communication channels and collaborative relationships between organizations will likely flourish. Specifically, the toolkit is designed to ensure regular, timely feedback that supports continued learning from experience, including learning from mistakes and unintended consequences. It is also designed to promote accountability. Effective, fluid communication, trust building, diplomacy, and networking are each critical to achieving project outcomes and building local health systems.^30^

The careful design and implementation of project management tools, driven collaboratively by local PIs, PMs, and other study stakeholders, using continuous evaluation and improvement planning, has the potential to ensure successful research outcomes and achieve long-term research capacity-building goals in LMICs. These successes further strengthen the generation of knowledge to inform public health needs in LMICs. Effective implementation of the toolkit described here occurred through cycles of dissemination and feedback, as facilitated by the PMWG. Investment in PM resources is essential for strengthening health research outcomes in LMICs.

## References

[1] Chu KM, Jayaraman S, Kyamanywa P (2014). Building research capacity in Africa: Equity and global health collaborations. PLoS Med.

[2] Yeager VA, Bertrand J (2015). Putting management capacity building at the forefront of health systems strengthening: Comment on “Management Matters: A Leverage Point for Health Systems Strengthening in Global Health”. Int J Health Policy Manag.

[3] Royston G (2011). Meeting global health challenges through operational research and management science. Bull World Health Organ.

[4] Bradley EH, Taylor LA, Cuellar CJ (2015). Management matters: A leverage point for health systems strengthening in global health. Int J Health Policy Manag.

[5] Marchal B, Cavalli A, Kegels G (2009). Global health actors claim to support health system strengthening: Is this reality or rhetoric?. PLoS Med.

[6] Swanson RC, Bongiovanni A, Bradley E (2010). Toward a consensus on guiding principles for health systems strengthening. PLoS Med.

[7] White F (2002). Capacity-building for health research in developing countries: A manager’s approach. Rev Panam Salud Publica.

[8] Garvin DA (1993). Building a learning organization. Harv Bus Rev.

[9] Persaud DD (2014). Enhancing learning, innovation, adaptation, and sustainability in health care organizations: The ELIAS performance management framework. Health Care Manag (Frederick).

[10] Roberts DW (2013). Improving care and practice through learning health systems. Nurs Manage.

[11] Swanson RC, Cattaneo A, Bradley E (2012). Rethinking health systems strengthening: Key systems thinking tools and strategies for transformational change. Health Policy Plan.

[12] Tashobya CK, da Silveira VC, Ssengooba F (2014). Health systems performance assessment in low-income countries: Learning from international experiences. Global Health.

[13] Adam T, de Savigny D (2012). Systems thinking for strengthening health systems in LMICs: Need for a paradigm shift. Health Policy Plan.

[14] Mutale W, Bond V, Mwanamwenge MT (2013). Systems thinking in practice: The current status of the six WHO building blocks for health system strengthening in three BHOMA intervention districts of Zambia—A baseline qualitative study. BMC Health Serv Res.

[15] Odedina FT, Akinremi TO, Chinegwundoh F (2009). Prostate cancer disparities in black men of African descent: A comparative literature review of prostate cancer burden among black men in the United States, Caribbean, United Kingdom, and West Africa. Infect Agent Cancer.

[16] MADCaP Network Project manager toolkit.

[17] National Institute of Dental and Craniofacial Research Toolkit & educational materials.

[18] National Heart, Lung, and Blood Institute Study quality assessment tools.

[19] MADCaP Network Men of African Descent and Carcinoma of the Prostate.

[20] Hopkins J, Elizabeth B, Tim E (2013). International twinning partnerships: An effective method of improving diagnosis, treatment and care for children with cancer in low-middle income countries. J Cancer Pol.

[21] MADCaP Network MADCaP study project manager roles survey.

[22] MADCaP Network MADCaP training videos.

[23] Wenger EMR, Snyder W Cultivating Communities of Practice: A Guide to Managing Knowledge.. Harvard Business School Press, Cambridge, MA. 2002:pp. 304.

[24] MADCaP Network Quality control/management plan.

[25] MADCaP Network Risk log assessment and follow up plan.

[26] MADCaP Network Enrollment status report.

[27] De Ceukelaire W, De Vos P, Criel B (2011). Political will for better health, a bottom-up process. Trop Med Int Health.

[28] IT Cortex Failure causes.

[29] Forjuoh SN, Helduser JW, Bolin JN (2015). Challenges associated with multi-institutional multi-site clinical trial collaborations: Lessons from a diabetes self-management interventions study in primary care. J Clin Trials.

[30] Atun R, Kazatchkine M (2009). Promoting country ownership and stewardship of health programs: The global fund experience. J Acquir Immune Defic Syndr.

